# An integrated analysis of microRNAs regulating DNA damage response in triple-negative breast cancer

**DOI:** 10.1007/s12282-023-01477-y

**Published:** 2023-06-21

**Authors:** Raviprasad Kuthethur, Maria Sona Jerome, Yashwanth Subbannayya, Sanjiban Chakrabarty

**Affiliations:** 1 Department of Cell and Molecular Biology, Manipal School of Life Sciences, Manipal Academy of Higher Education, Manipal, Karnataka 576104 India; 2 Centre of Molecular Inflammation Research (CEMIR), Department of Clinical and Molecular Medicine (IKOM), Norwegian University of Science and Technology, 7491 Trondheim, Norway; 3 School of Biosciences, Faculty of Health and Medical Sciences, University of Surrey, Guildford, GU2 7XH UK; 4 Center for DNA Repair and Genome Stability (CDRGS), Manipal Academy of Higher Education, Manipal, Karnataka 576104 India

**Keywords:** Homologous recombination, DNA repair, Triple-negative breast cancer, MicroRNA, Biomarkers

## Abstract

**Background:**

Triple-negative breast cancer (TNBC) remains a clinical challenge due to its aggressive phenotype and limited treatment options for the patients. Many TNBC patients show an inherent defect in the DNA repair capacity primarily by acquiring germline mutations in *BRCA1* and *BRCA2* genes leading to Homologous Recombination Deficiency (HRD). Epigenetic modifications such as *BRCA1* promoter methylation and miRNA expression targeting DNA repair pathway genes have contributed to the HRD phenotype in TNBC. Hence, we aimed to identify microRNAs that are associated with HRD status in the TCGA-BRCA project.

**Materials and methods:**

We implemented a miRNA prediction strategy for identifying miRNAs targeting HR pathway genes using an in silico predicted and experimentally validated list from published literature for their association with genomic instability and factors affecting HRD. In silico analysis was performed to study miRNA expression patterns regulated by DNA methylation and TMB status in the TNBC patients from TCGA-BRCA project. Finally, we analysed selected miRNA expression with immune cell infiltration pattern in the TNBC patient cohort.

**Results:**

Our study identified miRNAs associated with HRD, tumour mutation burden (TMB), and immune cell infiltration. Identified miRNA signatures were associated with the miR-17 ~ 92 cluster, miR-106b ~ 25 cluster, and miR-200b ~ 429 cluster. Pathway analysis of selected miRNAs suggested their association with altered immune cell infiltration in TNBC.

**Conclusion:**

Our study identified 6 ‘HRD associated miRNAs’ such as miR-106b, miR-93, miR-17, miR-20a, miR-200b, and miR-429 as novel miRNA-based signatures associated with HR deficiency in TNBC.

**Supplementary Information:**

The online version contains supplementary material available at 10.1007/s12282-023-01477-y.

## Introduction

Triple-negative breast cancer (TNBC) is a highly aggressive form of breast cancer that shows a worse prognosis than the other subtypes. It represents around 15–20% of breast cancer cases and is characterised by the absence of Estrogen (ER) and Progesterone (PR) hormone receptor expression, and HER2 gene amplification [1]. It is reported that the 5-year survival rate of TNBC patients is 76.9%, however, in metastatic TNBC, the survival rate is drastically reduced to 12% [1]. TNBC poses both a diagnostic and therapeutic challenge. TNBCs are difficult to identify using traditional mammography due to differences in tissue density and are often diagnosed at a more advanced stage [2]. Due to the absence of hormonal receptors, TNBC patients do not benefit from classical endocrine therapies and standardised regimens, with chemotherapy as a viable option. Accumulation of DNA damage leads to genomic instability, an enabling characteristic due to the presence of *BRCA1*/2 mutations leading to TNBC progression [3]. Thus, platinum-based DNA damaging drugs have shown more promising therapy response in TNBC patients. However, platinum-based regimens do not significantly improve in the progression-free survival (PFS) rate of metastatic TNBC [4]. In this regard, use of poly (ADP-ribose) polymerase inhibitors (PARP*i*) in combination with platinum-based therapies have shown pathological complete response (pCR) of up to 90% during phase 2 clinical trials [5–7]. However, identifying functional homologous recombination deficiency (HRD) is very important prior to the selection of eligible patients for PARP*i* therapy, to avoid the development of the tumour recurrence. Hence, TNBC patients would primarily benefit from the identification of early diagnostic biomarkers and therapeutic targets.

Interestingly, non-coding RNAs have been known to be dysregulated during various pathophysiological conditions, including cancer. MicroRNAs (miRNAs) are non-coding RNAs that are typically 17–23 nucleotides in length and function in post-transcriptional gene regulation. Previous studies have shown the dysregulation of miRNAs in cancer, contributing to drug resistance, tumour proliferation and growth, invasion, metastasis, angiogenesis, and apoptosis [8, 9]. Several miRNAs have been identified as biomarkers for the early diagnosis and prognosis of cancers [10]. Their dysregulation can occur due to the amplifications or deletions in miRNA genes, epigenetic changes such as methylation and histone modifications, altered transcriptional control and defects in microRNA biogenesis and processing [9, 11]. The stability of the miRNAs and relative ease of detection in body fluids makes them an attractive candidate as a minimally invasive marker for diagnosis, prognosis, and subtype identification [9]. In addition, miRNA-based therapeutics are on the rise, which involve replenishing tumour suppressor miRNAs or using miRNA antagonists to target oncogenic microRNAs [9].

Our study aims to identify miRNA signatures dysregulated during TNBC progression and associated with HR deficiency in TNBC patients. We identified miRNAs targeting HR pathway genes through in silico prediction, followed by their expression analysis in the TNBC cohort categorised into HR-proficient and -deficient TNBC to establish a signature to be validated and utilised for the patient stratification for PARP*i* therapy.

## Materials and methods

### MicroRNA-target gene prediction

MicroRNAs targeting HR pathway genes were identified with the use of three target prediction databases, miRDB [12], TargetScan [13], and TarBase v.8 [14]. The top 10% of predicted microRNAs for each HR gene from the three databases were selected. The microRNAs predicted by at least two of the three databases were shortlisted for further analysis. Similarly, miRTarBase v.8.0. was used to download experimentally validated target gene network, especially through dual-luciferase reporter assay [15]. Additionally, the target prediction tools were also used to identify HR targets of the top 20 upregulated and downregulated microRNA in TNBC from TCGA dataset. (https://portal.gdc.cancer.gov/projects/TCGA-BRCA).

### Expression analysis of microRNAs and target genes

The RNA-seq gene expression of the microRNAs and the target genes were analysed in the TCGA PanCancer Atlas breast cancer dataset for the TNBC cases. Gene and miRNA expression data was downloaded using the TCGA-Assembler tool and differential expression analysis was performed using DESeq2 package [16, 17]. Assignment of TCGA samples to TNBC subtypes was taken from Lehmann et al. [18, 23]. Gene expression of breast cancer cell lines was downloaded from Cancer Cell Line Encyclopaedia. Pearson correlation coefficients of miRNA–target gene pairs were computed using the miRNA reads per million and normalised read counts of the target genes. Survival analysis of the TNBC patients with respect to the expression status of individual miRNAs was performed using TCGA datasets.

### Methylation analysis of miRNA genes

Human Methylation 450 k beta values for 123 TNBC samples and 97 adjacent normal samples were downloaded from the TCGA-BRCA dataset. Methylation levels of the microRNAs in normal and tumour samples were analysed using the probes mapped to the respective genomic coordinates. The online tool, MethHC (https://awi.cuhk.edu.cn/~MethHC/), was also used to analyse the methylation levels of the microRNA gene promoters in normal versus tumour sampled in breast invasive carcinoma.

### Analysis of homologous recombination deficiency (HRD) status

The HRD scores of TCGA-BRCA samples were obtained from Takaya et al. (2020) [19]. The HRD score was calculated as the sum of the individual scores for loss of heterozygosity (LOH), telomeric allelic imbalance (TAI), and large-scale state transitions (LST). A HRD score of ≥ 42 was taken as the threshold for defining homologous recombination deficiency as determined by Telli et al. (2016) for TNBC samples [20].

### Tumour mutation burden (TMB)

Clinical data of breast cancer samples were downloaded from TCGA, having tumour mutation burden among breast cancer patients. We used median values to define TMB status of breast cancer patients as described previously [21].

### Correlation analysis of immune cell infiltration in TNBC with HR pathway-associated miRNA expression

TCGA patient IDs established as TNBC subtypes by Kalecky [22] and Lehmann et al*.* [23] were used for the analysis. We also downloaded TCGA patient IDs established as normal breast samples. We fetched immune cell quantitation for TNBC and adjacent normal breast patient IDs from TCGA using pre-calculated scores from TIMER 2.0 (http://timer.comp-genomics.org/, accessed 20th May 2022). We considered the CIBERSORT-abs scores for comparison as they allow for inter-sample comparison [24]. CIBERSORT uses arbitrary units that reflect the absolute proportion of immune cells in a mixture. A higher score indicates a higher proportion of immune cells [24]. We also downloaded level 3 Illumina HiSeq miRNA expression for all TCGA breast cancer samples using Firebrowse (firebrowse.org, accessed 20th May 2022) and filtered out expression values for TNBC and adjacent normal breast TCGA samples. Correlation between immune cell quantitation and miRNA expression data was carried out using in-house R scripts.

### Pathway analysis of select HR pathway miRNA candidates

To analyse the functional importance of select miRNAs in TNBC, we used miRTarBase 2022, an experimentally validated microRNA-target interaction database [15]. The miRTarBase database (https://mirtarbase.cuhk.edu.cn/~miRTarBase/miRTarBase_2022) was downloaded (Database accessed on May 16th, 2022) and filtered for human entries. The database was then queried for differential miRNAs. The target genes were then subjected to hypergeometric enrichment-based gene ontology and pathway analysis using Enrichr’s Reactome 2016 database (https://maayanlab.cloud/Enrichr/).

### Statistical analysis and figure generation

Statistical analysis for differential expression of miRNAs and target genes was performed using non-parametric two-tailed t-test (Mann–Whitney). *P* value of < 0.05 was considered as statistically significant.

## Results

### Identification of miRNAs targeting HR pathway genes

**Fig. 1:**
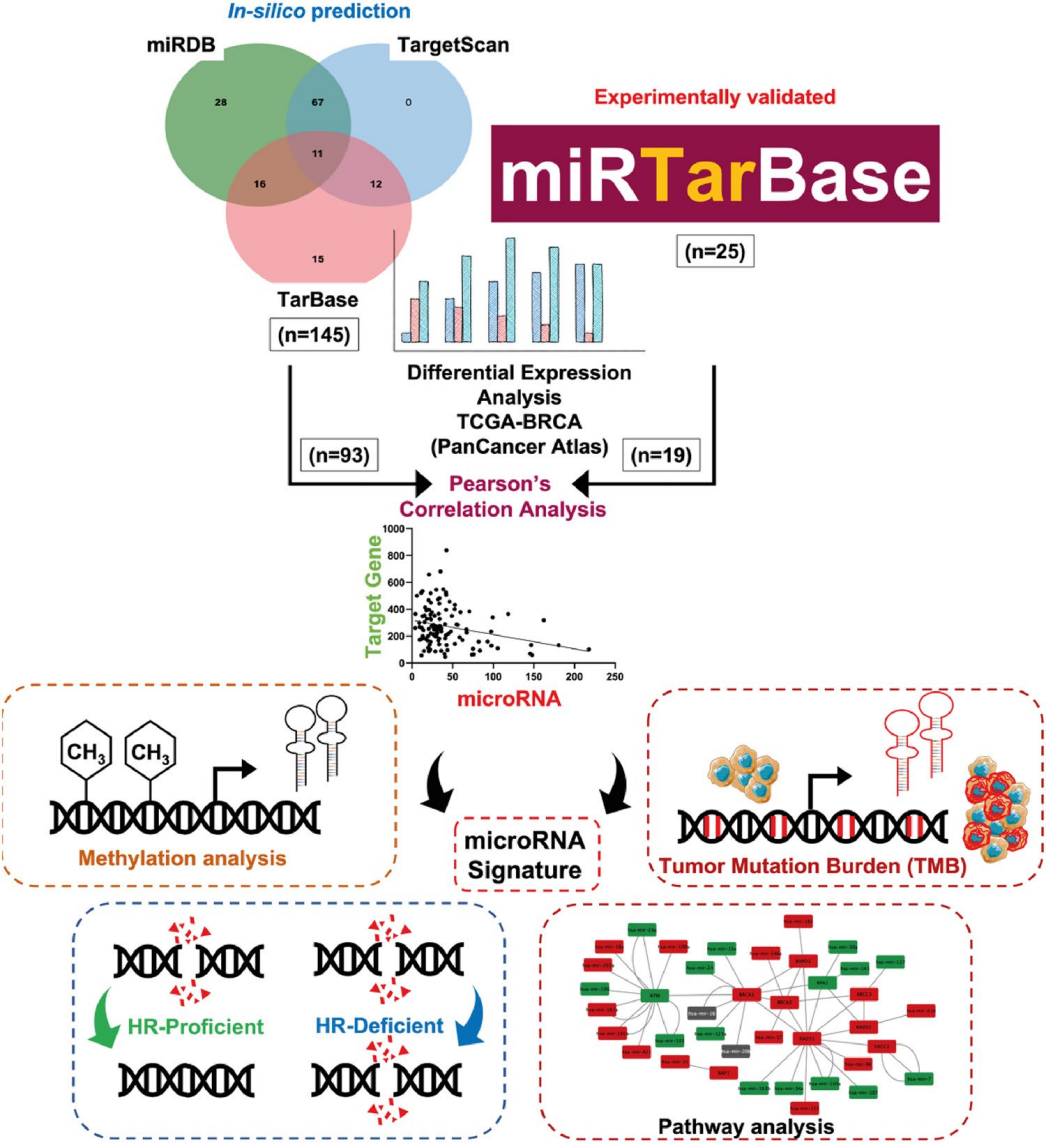
Schematic work flow of the study: the work flow comprises (i) miRNA prediction involving in silico and validated list from the miRNA:target gene interaction databases; (ii) differential expression analysis between normal and TNBC sample cohort from TCGA-BRCA PanCancer Atlas; (iii) miRNA/target gene expression correlation analysis; (iv) validation of the identified miRNA signature using methylation, TMB, HR-deficiency score, and pathway analysis

**Fig. 2:**
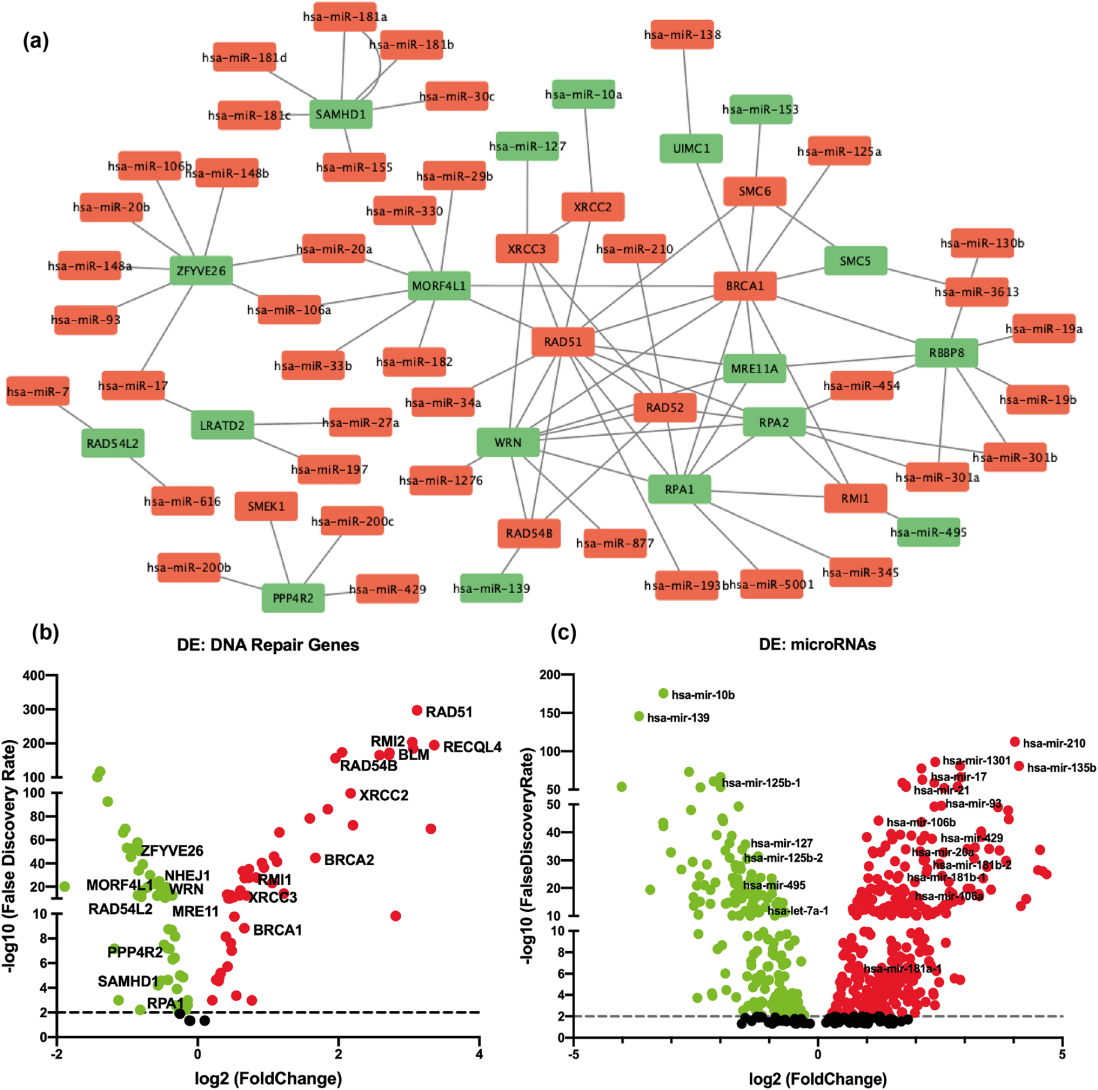
Differential expression analysis of identified miRNAs and HR pathway genes: **a** identified miRNA/HR pathway target gene network coupled with experimentally validated STRING interaction analysis. **b** and **c** Differential expression analysis of miRNAs and DNA repair pathway genes among TNBC sample cohort compared to normal samples. False discovery rate (FDR) was calculated from adjusted P value

To identify miRNAs targeting HR pathway genes, we downloaded the list of experimentally validated miRNAs targeting HR pathway genes from miRTarBase release 8.0 and shortlisted only those miRNA/target gene pairs that are validated using Luciferase reporter assay [15]. 25 miRNAs targeting 8 HR pathway genes were obtained (Table 1). In addition, we performed in silico target prediction using three independent targeting miRNA prediction tools: miRDB [12], TargetScan [13], and TarBase v.8 [14] tools to further analyse unidentified putative targeting miRNAs. Top 10% targeting miRNAs of all the HR pathway genes were selected and targeting miRNAs common in more than 2 target prediction tools were selected for further analysis (Fig. 1). In total, 149 miRNAs targeting 37 HR pathway genes were identified. Potential HR pathway gene targeting miRNAs interaction networks from both these approaches coupled with STRING interaction network were visualised using Cytoscape v.3.9.1 (Fig. 2a).

**Table 1:** Validated list of microRNAs targeting HR pathway genes

Sl. no	HR pathway genes	Description	Targeting microRNAs (Validated)
1	*BRCA1*	Plays central role in the HR by forming complex with BRCA2 and RAD51 proteins	hsa-miR-146a, hsa-miR-146b, hsa-miR-16, hsa-miR-15a, hsa-miR-24, hsa-miR-20b, hsa-miR-125a, hsa-miR-182
2	*BRCA2*	Plays central role in the HR by forming complex with BRCA1 and RAD51 proteins	hsa-miR-146a, hsa-miR-17, miR-19a, miR-19b
3	*RAD51*	Recruited at DSB sites to facilitate HR along with BRCA1/2 and other RAD51 family proteins	hsa-miR-96, hsa-miR-193b, hsa-miR-103a, hsa-miR-155, hsa-miR-107, hsa-miR-34a
4	*RAD52*	Binds to DNA nicks at DSB sites to recruit BRCA1/2 and RAD51 complex proteins	hsa-miR-210, miR-373
5	*RPA1*	Stabilises ssDNA intermediates	hsa-miR-145, hsa-miR-30a
6	*SAMHD1*	DNA end resection	hsa-miR-181a, hsa-miR-155
7	*XRCC2*	Binds to double strand breaks and facilitates HR	hsa-miR-7
8	*XRCC3*	Binds to double strand breaks and facilitates HR	hsa-miR-127
9	*XRCC5*	Binds to double strand breaks and facilitates HR	hsa-miR-139
10	*RAD54L*	Facilitates homologous pairing	hsa-miR-139

### Expression analysis of miRNAs and their HR pathway gene targets

**Fig. 3:**
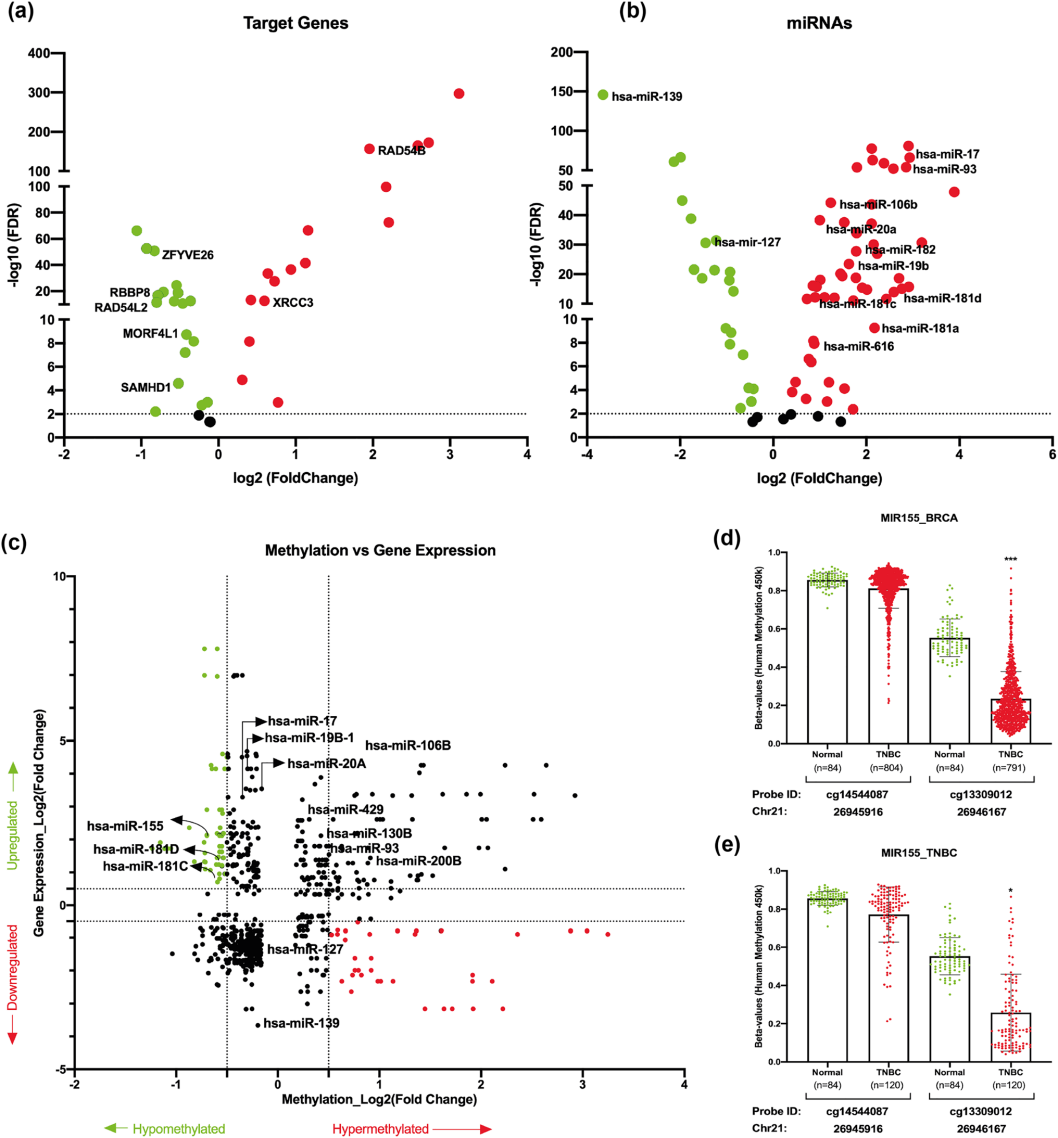
miRNA/target genes and methylation analysis of signature miRNAs: **a** and **b** shortlisted miRNAs and their target genes upon filtering miRNAs based on their expression status in TNBC sample cohort. **c** Starburst plot showing integrated DNA methylation and gene expression data. **d** Methylation analysis of individual human methylation 450 k probes on promoter region of miR-155 from all breast cancer samples. **e** Methylation analysis of the same probes of miR-155 from TNBC samples. Unpaired (two-tailed) student’s *t* test was performed between each normal and tumour sample groups; *P value < 0.05, **P value < 0.001, and ***P value < 0.0001

Further, we performed differential expression analysis of shortlisted HR pathway genes and their targeting miRNAs in TNBC samples to validate their feasibility to be utilised as biomarkers (Fig. 2b). Differential expression analysis of the targeting miRNAs as well as HR pathway genes in TNBC samples from TCGA-BRCA PanCancer datasets filtered 93 miRNAs out of 145 miRNAs (Table S2). However, negative correlation analysis between miRNAs and target genes further filtered approximately 53% (50/93) of the targeting miRNAs as well as 50% (19/37) of the target genes with significant negative correlation from the in silico predicted list (Table S2). On the other hand, approximately, 76% (19/25) of the targeting miRNAs were filtered from the experimentally validated list upon differential expression analysis (Table S3). Finally, Pearson’s correlation analysis further filtered 13 miRNAs targeting 6 HR pathway genes with significant negative correlation upon comparison (Fig. 3a, b; Figure S1) (Table S4). Interestingly, most of the miRNAs in the signature were part of the miRNA clusters and/or families, where miR-106b and miR-93 are part of the miR-106b ~ 25 cluster (Figure S1a); miR-17 and miR-20a are part of the miR-17 ~ 92 cluster (Figure S1b); miR-181a, miR-181b, miR-181c, and miR-181d are part of miR-181 family (Figure S1c); miR-200b and miR-429 are part of the miR-200 family, cluster I (Figure S1d); with pathophysiological implications in various tumours [25]. Hence, these miRNA/target gene pairs with significant negative correlation were further selected for downstream analysis to validate their application as potential biomarkers.

### Analysis of upstream regulators of signature miRNAs in TNBC

We then analysed these miRNAs for epigenetic upstream regulating factors such as promoter DNA methylation to understand their regulation pattern in TNBC cohort. Combined differential methylation analysis and gene expression analysis identified miR-155, miR-181c, and miR-181d to be hypomethylated in breast cancer sample cohort compared to the normal sample cohorts (Fig. 3c). Since these miRNAs were upregulated in breast cancer cohorts (TCGA-BRCA), promoter hypomethylation pattern suggested significant correlation with their expression status. However, methylation analysis further in TNBC cohort of miR-155 showed corroborating hypomethylation pattern (Fig. 3d–e), but miR-181c and miR-181d did not show any differential methylation signature among TNBC cohort compared to the normal sample cohort (Figure S2).

### Validation of miRNA signature for tumour mutation burden (TMB)

**Fig. 4:**
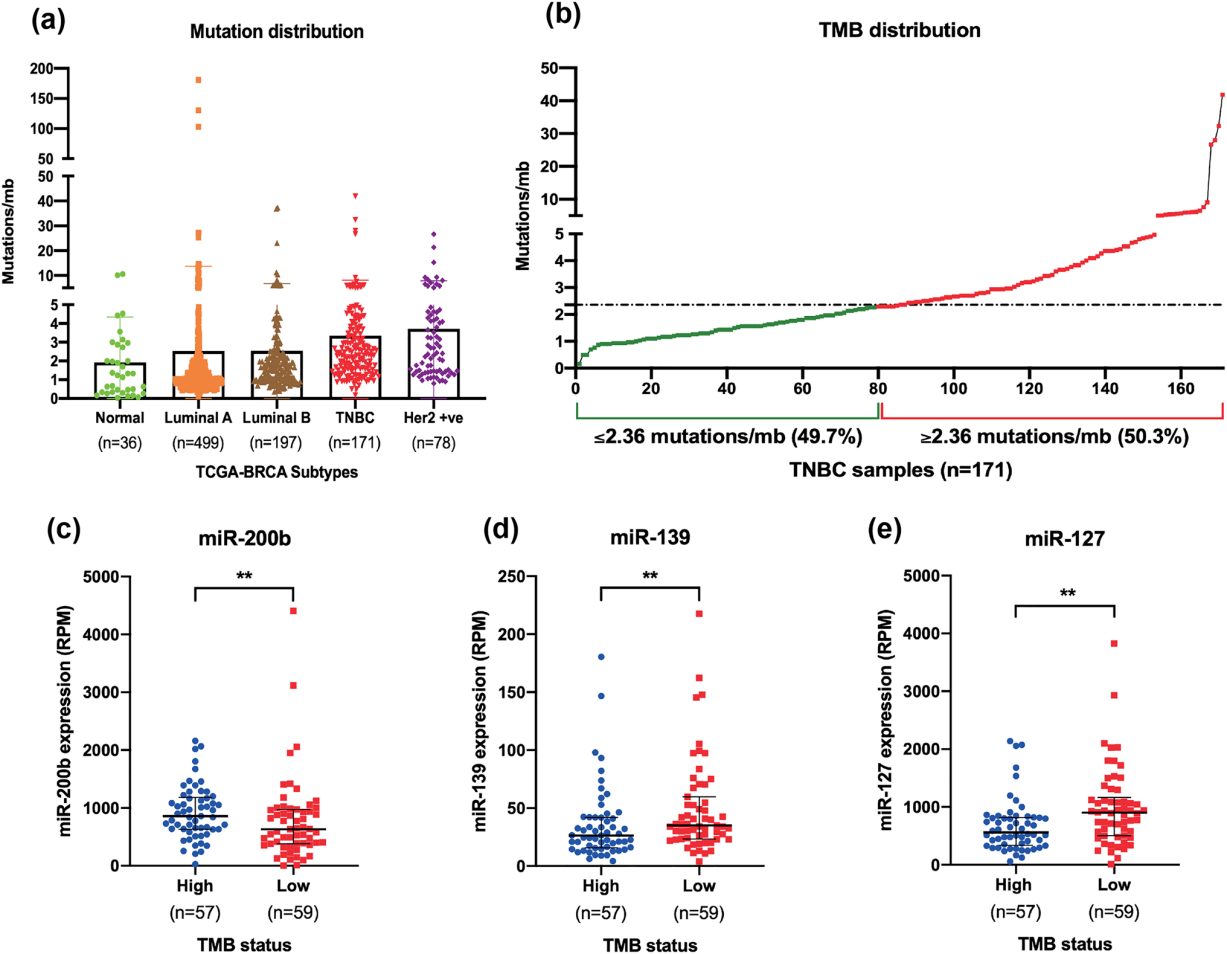
TMB analysis of signature miRNAs: **a** mutation count distribution among normal and different subtypes of breast cancer subtypes. **b** TMB distribution based on median threshold among TCGA-BRCA PanCancer datasets. Expression analysis of signature miRNAs among TMB-high and -low samples for **c** miR-200b, **d** miR-139, and **e** miR-127. Unpaired (two-tailed) student’s *t* test was performed between high and low group TMB samples; **P value < 0.001

We then assessed genomic instability hallmarks such as TMB to validate its correlation with signature miRNAs [26]. TMB is the number of nonsynonymous somatic mutations per mega base of genomic region interrogated, which can be extrapolated to genome instability and acts as a predictive clinical biomarker for immune checkpoint inhibitor therapy [27]. Interestingly, mutation distribution analysis among different subtypes of TCGA breast cancer cohorts showed notable increase in the overall mutation count of TNBC and Her2 positive subtypes (Fig. 4a). However, TMB status in the overall TCGA-BRCA PanCancer cohort varied from 0 to 180, where 29 out of 1084 samples (2.67%) showed ≥ 10 mutations/mb sequence, suggesting significant negative skew distribution (Figure S3a). Hence, we used a different approach to classify the samples as TMB-high and TMB-low based on the median cutoff as described previously [21]. TMB count among TNBC samples varied from 0.16 to 41, where median was found to be 2.36. Hence, we sorted the TCGA-TNBC samples as TMB-High and TMB-Low based on the median distribution and analysed the expression status of signature miRNAs (Fig. 4b). miR-139 and miR-127 which are downregulated in TNBC cohort were associated with the TMB-low group, whereas miR-200b was associated with TMB-high group (Fig. 4c–e). Consistently, miR-139 was previously shown to be negatively associated with breast cancer progression and known to induce genomic instability by targeting essential DNA repair genes [28]. Similarly, miR-127 was negatively associated with TNBC progression and increased TNBC cell sensitivity to chemotherapy agents such as doxorubicin and cisplatin [29]. However, miRNAs from miR-106b ~ 25 cluster, miR-17 ~ 92 cluster, miR-181 family, and other miRNAs such as miR-429, miR-182 expression were not associated with differential TMB cohorts of TNBC samples (Figure S3b).

### Validation of miRNA signature among HR-proficient and -deficient TNBC

**Fig. 5:**
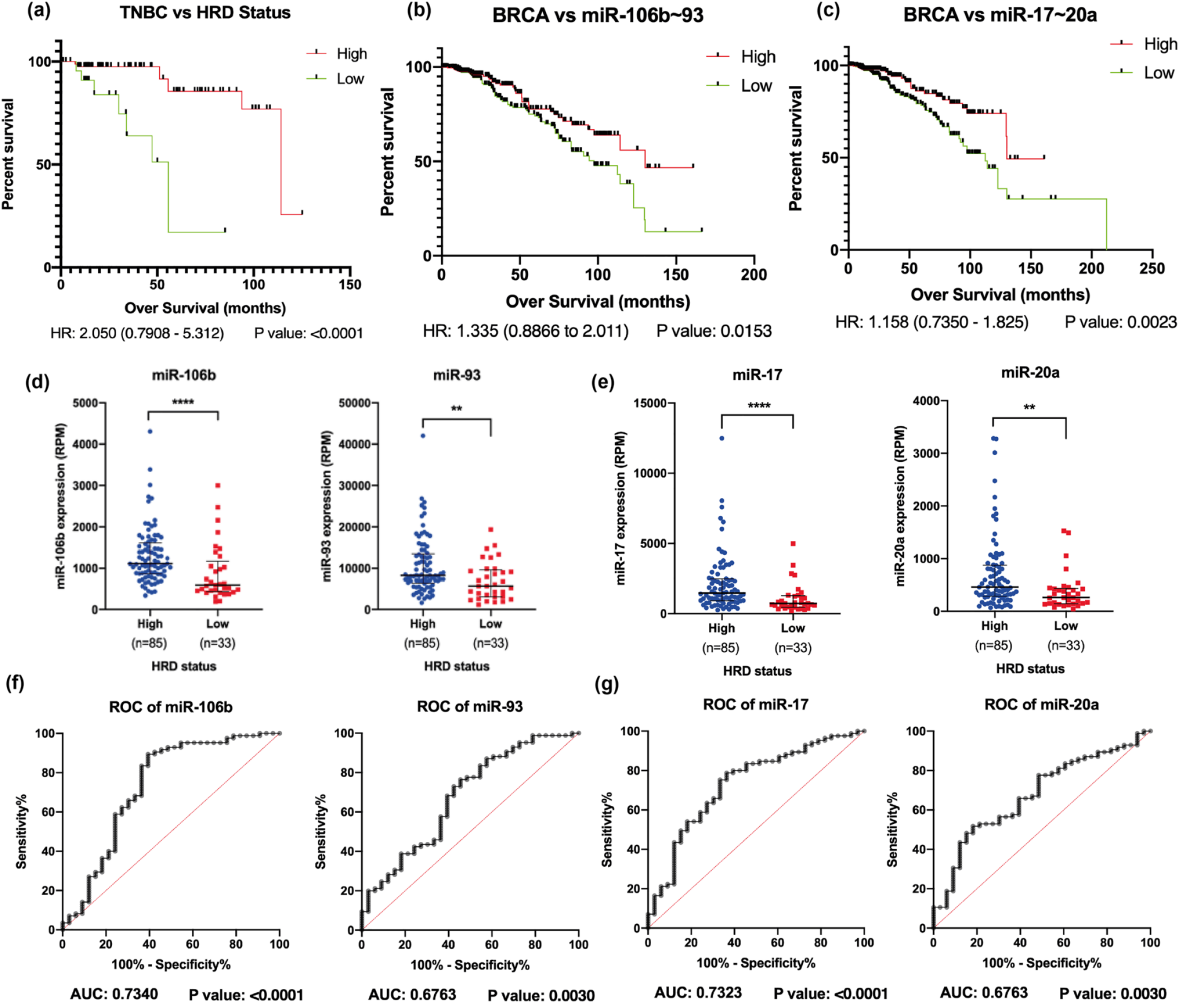
HRD and pathway analysis of signature miRNAs: **a** survival analysis of TNBC patients with high and low HRD status. **b** Survival analysis of BRCA patients with high and low levels of miR-106b and miR-93. **c** Survival analysis of BRCA patients with high and low levels of miR-17 and miR-20a. Expression analysis of signature miRNAs among high and low levels of HRD TNBC sample cohorts for **d** miR-106b and miR-93, **e** miR-17 and miR-20a. Unpaired (two-tailed) student’s *t* test was performed between high and low group HRD samples; **P value < 0.001 and ***P value < 0.0001. ROC curve analysis for analysing the diagnostic potentials of signature miRNAs **f** miR-106b and miR-93, **g** miR-17 and miR-20a

**Fig. 6:**
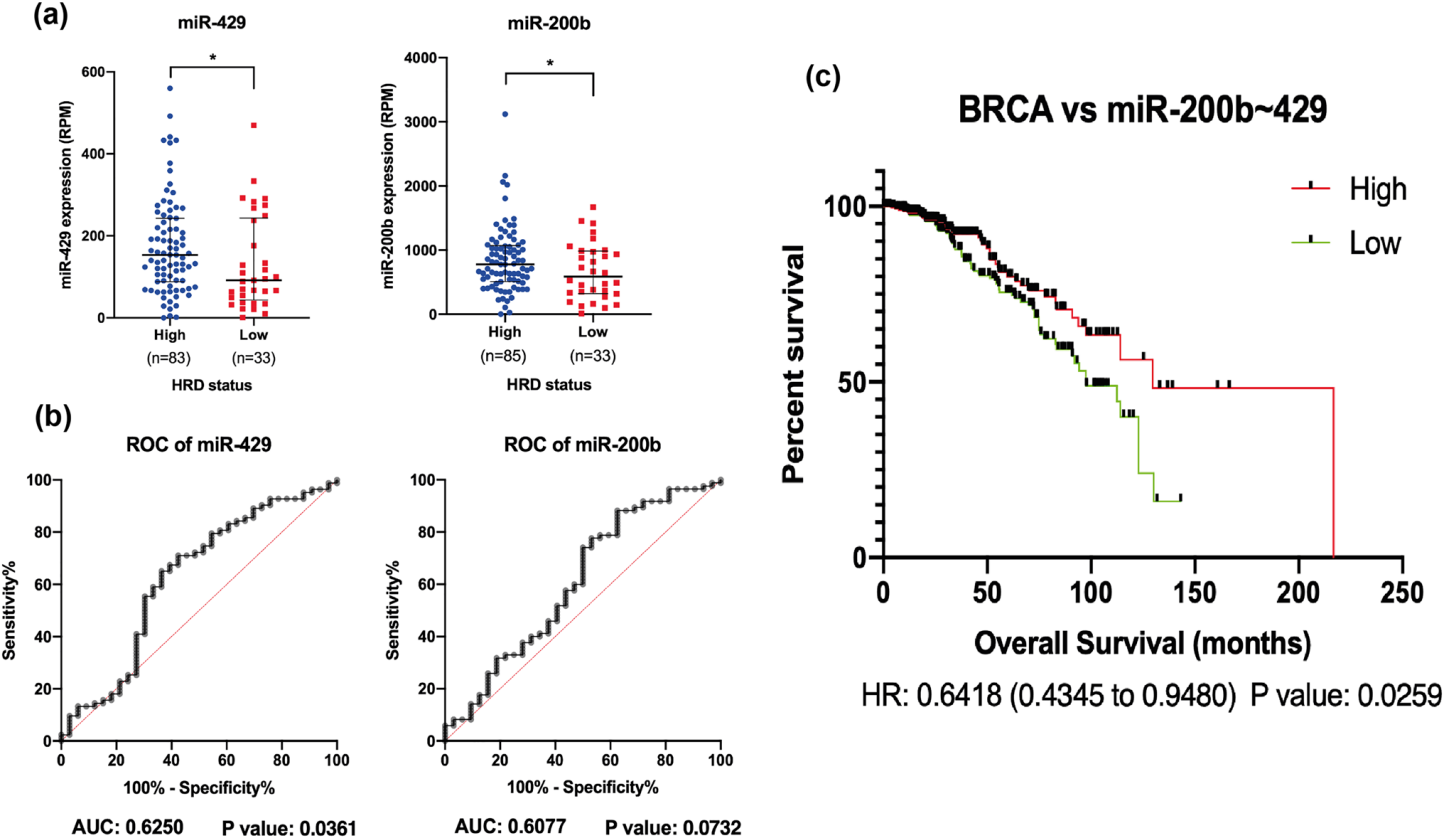
HRD and pathway analysis of signature miRNAs: **a** expression analysis of signature miRNAs among high and low levels of HRD TNBC sample cohorts for miR-429 and miR-200b. Unpaired (two-tailed) student’s *t* test was performed between high and low group HRD samples; *P value < 0.05. **b** ROC curve analysis for analysing the diagnostic potentials of signature miRNAs, miR-429 and miR-200b. **c** Survival analysis of BRCA patients with high and low levels of miR-200b and miR-429

Further, we assessed the association of our signature miRNAs with HRD to identify the efficient biomarkers for stratifying patients prior to PARP*i* therapy among TNBC cells. We calculated HRD score as described previously for TNBC samples among breast cancer cohort [19, 20]. We first analysed the survival status of breast cancer patients with HRD-high and -low cohorts and observed patients with high HRD showed remarkably better survival probability compared to patients with low HRD (Fig. 5a). Further, we identified TNBC upregulated miRNAs from miR-106b ~ 25 cluster, miR-106b and miR-93, were found to be associated with better survival probability among breast cancer patients along with their positive correlation with HR deficiency (Fig. 5b and d). Similarly, miRNAs from miR-17 ~ 92 cluster, miR-17 and miR-20a were also found to be associated with high HR deficiency and better patient survival (Fig. 5c and e). ROC curve analysis of both the miRNA clusters showed significant sensitivity towards their ability to differentiate between HR-deficient and -proficient sample cohorts (Fig. 5f, g). Pathway analysis of target genes of both the clusters was enriched in different phases of cell cycle pathways and cellular responses to stress (Figure S4a-b). Similarly, miR-200b, and miR-429 showing positive association with high HRD group, showed significant sensitivity towards HR-deficient sample cohort and also associated with better patient survival (Fig. 6). While their pathway analysis suggested the association of target genes in cellular stress response and innate immunity pathways (Figure S4c), remaining miRNAs from miR-181 family and miR-182 did not show any change in expression in HRD-high and HRD-low groups from TNBC cohorts (Figure S5), whereas downregulated miRNAs miR-139 and miR-127 were found to be associated with low HRD group of TNBC cohort as well as poor survival probability among breast cancer patients (Figure S6). Hence, we shortlisted 6 miRNAs, miR-106b, miR-93, miR-17, miR-20a, miR-200b, and miR-429 as HRD responsive biomarkers among TNBC cohort. We also validated miRNA:mRNA interaction between signature miRNAs and their target genes and observed target genes involved key cellular pathways such as cell cycle, DNA repair, cell proliferation, migration, and apoptotic pathways (Figure S7).

### Association of differential miRNA expression in TNBC with immune cell infiltration

Finally, we assessed the association of HRD responsive biomarker miRNAs with target genes enriched in innate immunity with immune cell infiltration and compared their expression pattern. To achieve this, we filtered TCGA miRNA expression data for miR-106b, miR-93, miR-17, miR-20a, miR-200b, and miR-429 in TNBC and adjacent normal samples and correlated them with pre-calculated CIBERSORT-abs tumour infiltration scores from TIMER. Interestingly, higher expression of miR-200b and miR-429 in TNBC correlated with lower infiltration of CD8+ T cells, resting CD4+ memory T cells, and M2 macrophages in comparison with normal (Figure S8a, b). Further, higher expression of miR-106b correlated with lower infiltration of B plasma cells, CD8+ T cells and M2 macrophages in TNBC compared to normal while higher expression of miR-93 correlated with lower infiltration of B plasma cells and CD8+ T cells in TNBC compared to normal. Expression of miR-20a and miR-17 in TNBC did not show any significant correlations with immune cell infiltration. These findings indicate the potential association of these miRNAs—miR-200b, miR-429, mIR-106b, and miR-93 with immune signalling in TNBC (Figure S9).

## Discussion

Maintaining genomic stability is fundamental for cell survival which is primarily governed by coordinated action of the DNA damage response (DDR), DNA replication, DNA repair and cell cycle progression. Defective DDR, replication stress and chromatin modifications are some of the hallmarks of cancer cells, leading to development and progression of tumour malignancies, while this can also be exploited to develop patient stratification and to design novel precision cancer therapies [30]. Cancer cells with defective DDR often rely on other functioning DNA repair pathways for survival. Chemotherapeutic agents that target DDR mechanism can selectively kill cancer cells without killing normal cells that do not rely on these pathways thus providing efficient way of treating cancer. However, identifying patients with functional HR deficiency is crucial for selecting patients eligible for neoadjuvant PARP*i* therapy, to ensure no tumour recurrence through secondary mechanisms which lead to poor therapy response and recovery. Currently, there are few studies for identifying diagnostic biomarkers and prognostic models based on their gene expression patterns to ensure early diagnosis and precision therapy. Various groups have attempted the utilisation of molecular signatures involving DNA repair genes and miRNAs expression patterns to validate tumour prognosis and predict therapy response in ovarian cancer [31], hepatocellular cancer [32], colon cancer [33], oesophageal cancer [34], and colorectal cancer [35]. However, our study identifies a novel miRNA signature to stratify TNBC patients prior to neoadjuvant PARP*i* therapy to determine their eligibility.

MicroRNAs have been recently identified to target various DNA repair pathway genes mainly HR pathway to regulate tumour progression [36]. Their interaction plays crucial role in determining cellular response to various chemotherapeutic agents and radiation therapies by modulating the DNA damage repair proficiency of cancer cells [28, 37–40]. Identifying the right signature with significant differential expression pattern and better sensitivity in differentiating the HR-deficient TNBC cohort from proficient cohort are the main objectives in the establishment of miRNA signature. In this regard, we performed miRNA prediction of HR pathway genes using two different strategies: (i) in silico identification of miRNAs using 3 different web-based prediction tools; (ii) literature survey combined with experimentally validated miRNA–target gene interaction database, to short-list all the known and unknown potential miRNAs. This strategy combines the benefit of identifying all the yet-unknown miRNAs that could potentially target HR pathway genes along with already identified potential miRNA/target gene pairs to comprehensively analyse the whole network. STRING interaction analysis coupled miRNA/target gene network showed experimentally validated physical interaction status among the target genes. Further downstream validations were implemented to filter out false-positive interaction pairs and establish clinically relevant miRNA signature for TNBC cohort. Differential expression analysis of the identified miRNA/target gene pairs in TNBC cohort from TCGA-BRCA PanCancer Atlas datasets filtered majority of the false-positive miRNAs with inability to differentiate between normal and TNBC cohort. Further, stringent filter was implemented by statistical analysis involving Pearson correlation co-efficiency to identify significant negative correlation between signature miRNA and their respective target genes.

Various upstream factors such as genetic and epigenetic factors are known to regulate the expression of miRNAs in various phenotypes involving cancer. The expression status revolves around tissue specificity and pathophysiological role of the respective miRNAs in respective phenotypes [41]. Elucidating such factors are also necessary to understand the upstream regulating factors to correlate miRNA expression with their functions. Promoter DNA methylation is known to regulate miRNA expression based on the extent of methylation status acting as an upstream regulatory mechanism. However, differential methylation analysis combined with miRNA expression status filtered miR-155 to be hypomethylated in TNBC cohort compared to normal cohort. Interestingly, miR-155 was previously also shown to be hypomethylated and associated with low survival probability among anaplastic glioma patients [42]. However, miR-181c and miR-181d were regulated in total breast cancer patient cohort, but not in TNBC cohort.

Tumour mutation burden is an important factor recently proposed to be involved with genome instability and established as a predictive clinical biomarker for immune checkpoint inhibitor therapy [27]. Higher mutation rate per mega base of genomic region sequenced suggests higher mutation burden leading to enhanced genome instability in malignant tumours. miR-200b which is previously known to inhibit breast cancer cell progression and metastasis in TNBC cells are positively associated with TMB among TNBC cohort [43]. Further validation of these miRNAs in high and low HRD TNBC cohort showed positive association of miRNAs from miR-106b ~ 25 cluster, miR-106b and miR-93; miR-17 ~ 92 cluster, miR-17 and miR-20a; miR-200b ~ 429 cluster, miR-429 and miR-200b. Interestingly, these miRNAs are also previously shown to be associated with cell cycle regulation and DNA damage response [36]. TNBC patients with high HR deficiency showed better survival probability, mostly due to their higher response rate to the chemotherapeutic agents combined with PARP*i* therapy, suggesting the efficacy of the treatment regimen. Consistently, higher expression of all the miRNAs (miR-106b ~ 93, miR-17 ~ 92, and miR-200b ~ 429 cluster) with positive correlation to high HR-deficiency group showed better survival probability among TNBC patients suggesting their sensitivity and specificity to differentiate between responsive and non-responsive patient cohort. Our study also showed the correlation of miR-200b and miR-429 with immune cell infiltration phenotypes among normal and TNBC patient cohort, due to the enrichment of their target genes in innate immune response pathway along with cellular responses to stress and senescence.

Our study made an attempt to establish a clinically relevant HRD responsive miRNA signature to stratify patients eligible for neoadjuvant PARP*i* therapy, using miRNAs differentially expressed in HR-deficient and -proficient TNBC cohort. A detailed experimental study in a clinical cohort of TNBC patients will facilitate validation of our signature miRNA expression to confirm its utility in clinical application. Further, these signature miRNAs must be functionally validated in both in vitro and in vivo studies to be able to establish them as diagnostic ‘HRD regulating miRNA signature’ to benefit TNBC patients with HR deficiency.

## Supplementary Information

Below is the link to the electronic supplementary material.

Supplementary file1 (DOCX 4945 KB)

## Data Availability

All data generated or analysed during this study are included in this published article/Supplementary Material. Further inquiry can be directed to the corresponding author.

## Abbreviations

TNBC: Triple-negative breast cancer
HR: Homologous recombination
HRD: Homologous recombination deficiency
DSBs: Double-strand breaks
DDR: DNA damage response
TCGA: The Cancer Genome Atlas
TMB: Tumour Mutation Burden
PARP*i*: Poly (ADP-ribose) polymerase inhibitors
