# Supplementary material for: An integrated analysis of microRNAs regulating DNA damage response in triple-negative breast cancer
Source: Breast Cancer. 2023 Jun 21;30(5):832–44. doi: 10.1007/s12282-023-01477-y (PMC10404216; doi:10.1007/s12282-023-01477-y)
Supplement: Supplementary file 1 — Supplementary file1 (DOCX 4945 KB) [file 12282_2023_1477_MOESM1_ESM.docx]

**An integrated analysis of microRNAs regulating DNA damage response in triple-negative breast cancer**

Raviprasad Kuthethur^1^, Maria Sona Jerome^1^, Yashwanth Subbannayya^2,3^, Sanjiban Chakrabarty^1,4^*

^1^Department of Cell and Molecular Biology, Manipal School of Life Sciences, Manipal Academy of Higher Education, Manipal-576104, Karnataka, India

^2^Centre of Molecular Inflammation Research (CEMIR), Department of Clinical and Molecular Medicine (IKOM), Norwegian University of Science and Technology, 7491 Trondheim, Norway.

^3^School of Biosciences, Faculty of Health and Medical Sciences, University of Surrey, Guildford, GU2 7XH, UK

^4^Center for DNA Repair and Genome Stability (CDRGS), Manipal Academy of Higher Education, Manipal-576104, Karnataka, India

*Corresponding Author: Sanjiban Chakrabarty, Ph.D., Department of Cell and Molecular Biology, Manipal School of Life Sciences, Manipal Academy of Higher Education, Manipal, Karnataka 576106, India. E-mail: sanjiban.c@manipal.edu,

**Running title:** miRNAs targeting HR pathway in TNBC

**Figure S1:** Pearson correlation analysis between shortlisted miRNAs and their target genes: Upregulated miRNAs (a) miR-106b~25 cluster, (b) miR-17~20a/92 cluster, (c) miR-181 family, (d) miR-200b~429 cluster, and (e) miR-182. Downregulated miRNAs: (f) miR-139 and miR-127. miRNA/target genes upregulated are labelled in red whereas, miRNA/target genes downregulated are labelled in green.

**Figure S2:** (a) Methylation analysis of individual human methylation 450k probes on promoter region of miR-181c from all breast cancer samples. (b) Methylation analysis of the same probes of miR-181c from TNBC samples. (c) Methylation analysis of individual human methylation 450k probes on promoter region of miR-181d from all breast cancer samples. (d) Methylation analysis of the same probes of miR-181d from TNBC samples. Unpaired (two-tailed) student’s t-test was performed between each normal and tumour sample groups; *P-value<0.05, **P-value<0.001, and ***P-value<0.0001.

**Figure S3:** (a) TMB distribution based on standard ≥10 mutation/mb threshold among TCGA-BRCA PanCancer datasets. (b) Expression analysis of miRNA signature in median TMB breast cancer cohort (TNBC). TNBC samples were sorted based on non-synonymous TMB mutation count obtained from GDC data portal. Unpaired (two-tailed) student’s t-test was performed between high and low group TMB samples.


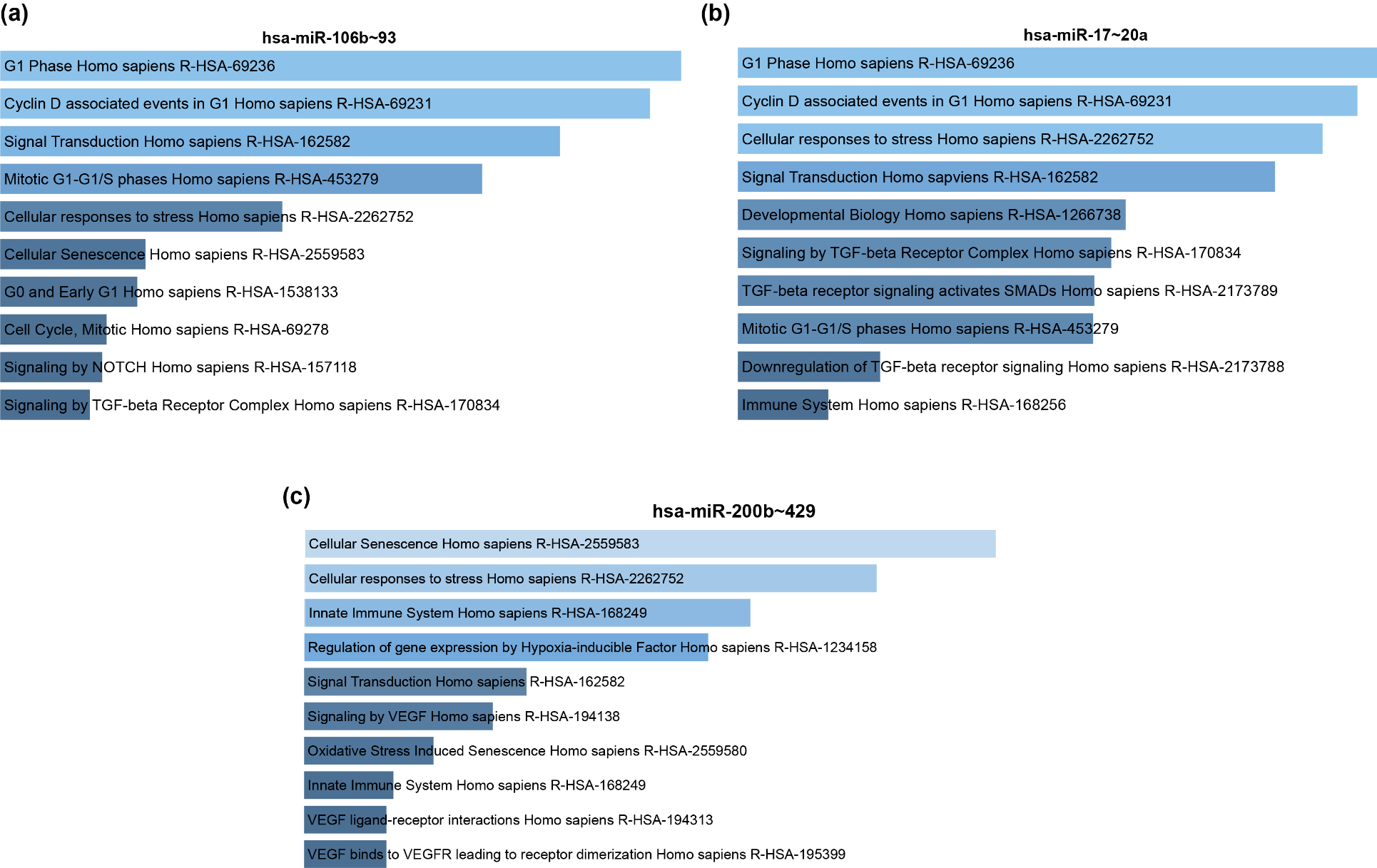


**Figure S4:** Reactome pathway analysis using Enrichr tool for target genes of (a) miR-106b~93 cluster; (b) miR-17~20a cluster; and (c) miR-200b~429 cluster, sorted based on P-value ranking (P<0.05).


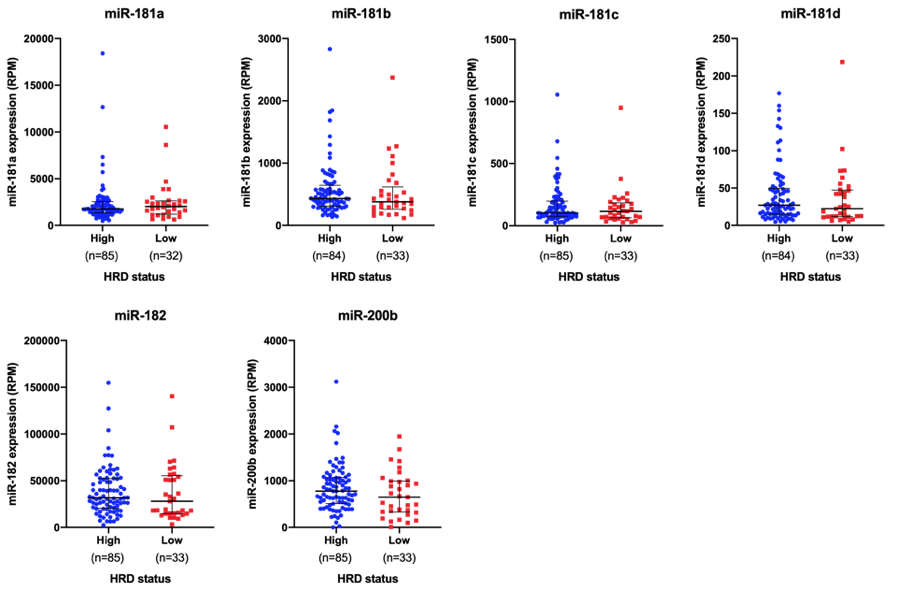


**Figure S5:** Expression analysis of miRNA signature in high and low HRD breast cancer cohort (TNBC). Unpaired (two-tailed) student’s t-test was performed between high and low group HRD samples.

**Figure S6:** (a) Expression analysis of miR-139 and miR-127 in high and low group HRD breast cancer cohort (TNBC). Unpaired (two-tailed) student’s t-test was performed between high and low group HRD samples. **P-value<0.001 and ***P-value<0.0001. (b) Survival analysis of breast cancer patients with high and low expression levels of miR-139. (c) Reactome pathway analysis for target genes of miR-139 and miR-127, sorted based on P-value ranking.


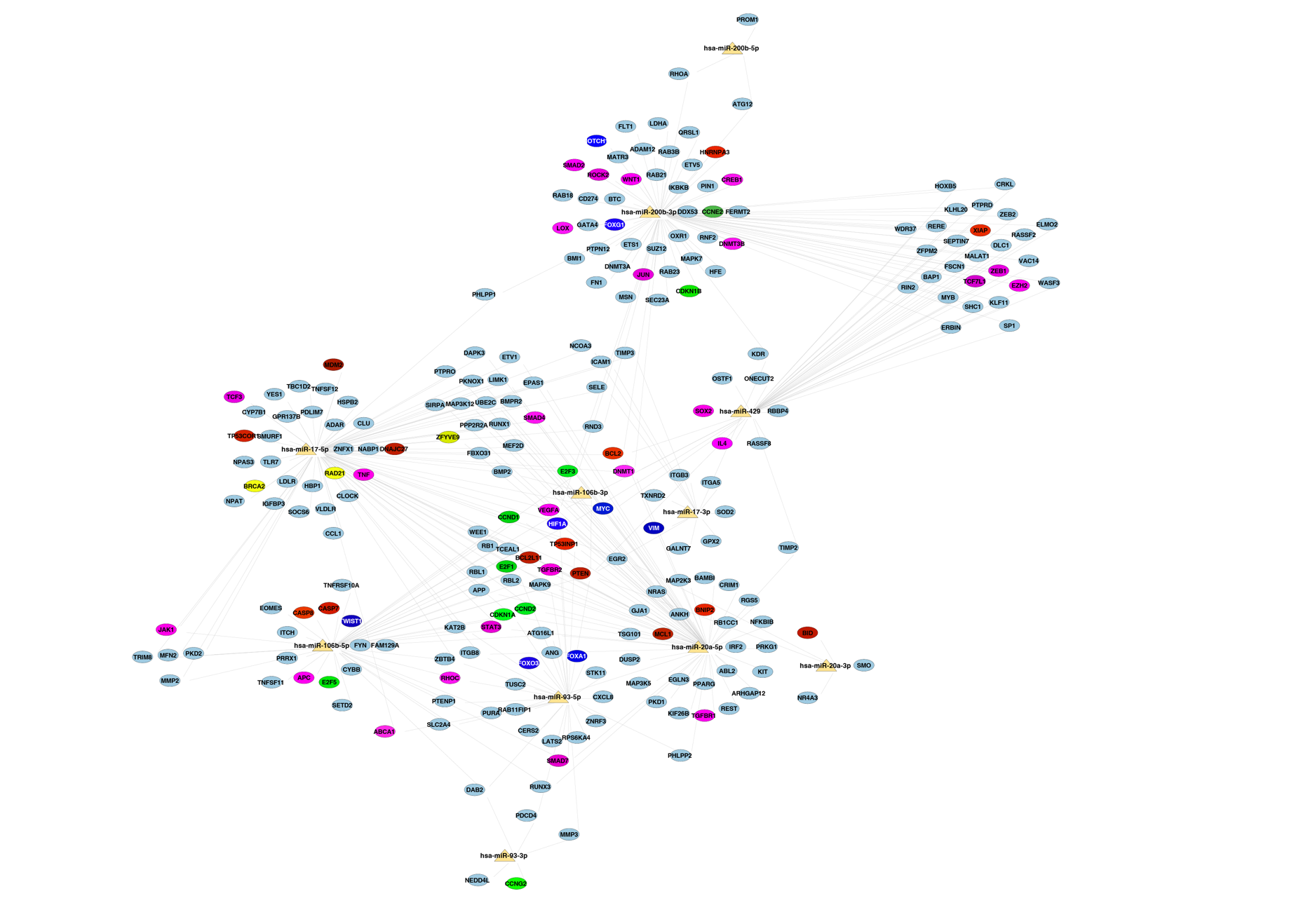


**Figure S7:** **miRNA: mRNA pathway interaction:** Target genes of signature miRNAs were analysed for pathway interaction. Target genes were involved in multiple key pathways such as cell cycle, DNA damage repair, cell proliferation, migration, and apoptosis etc.


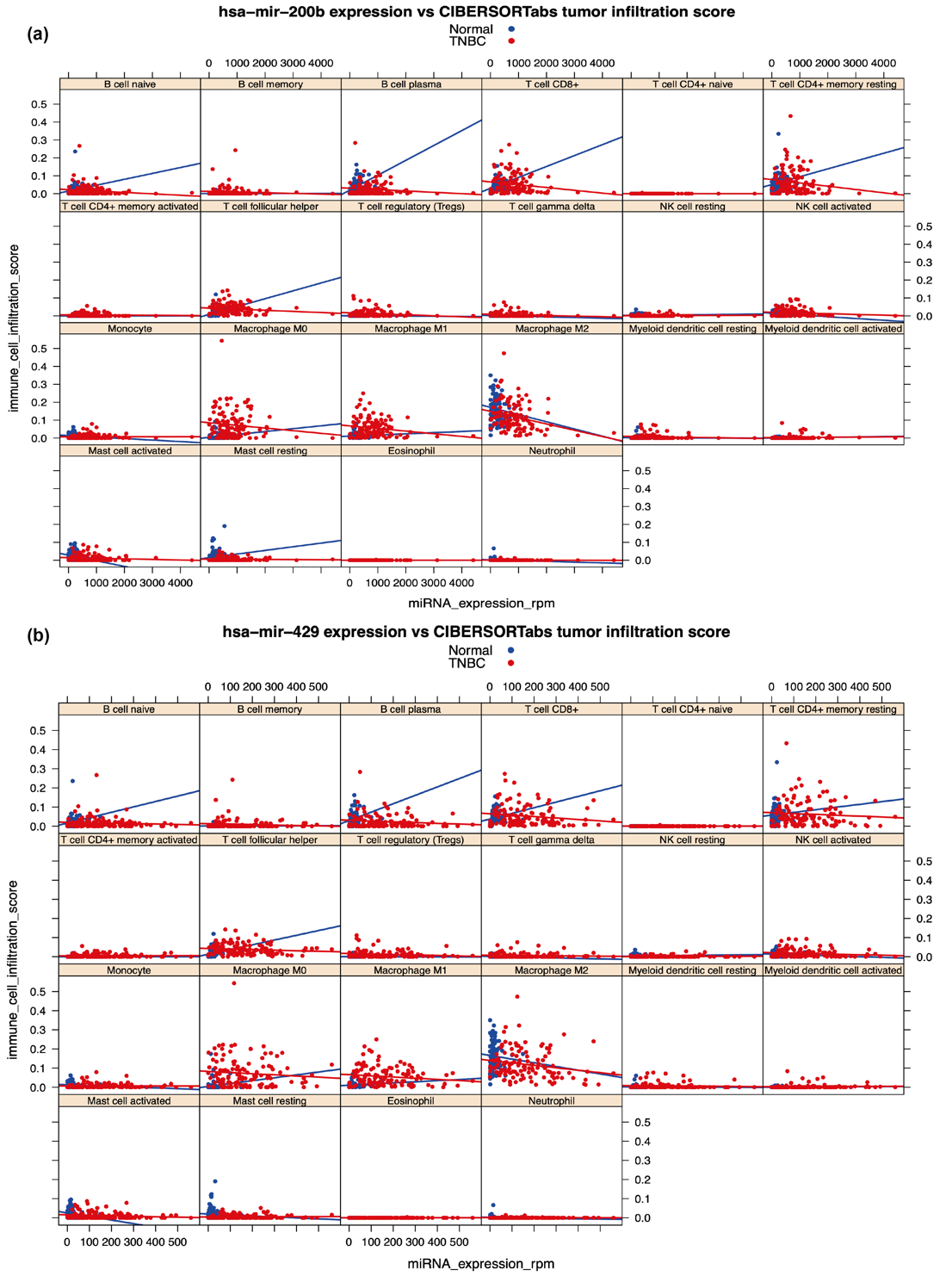

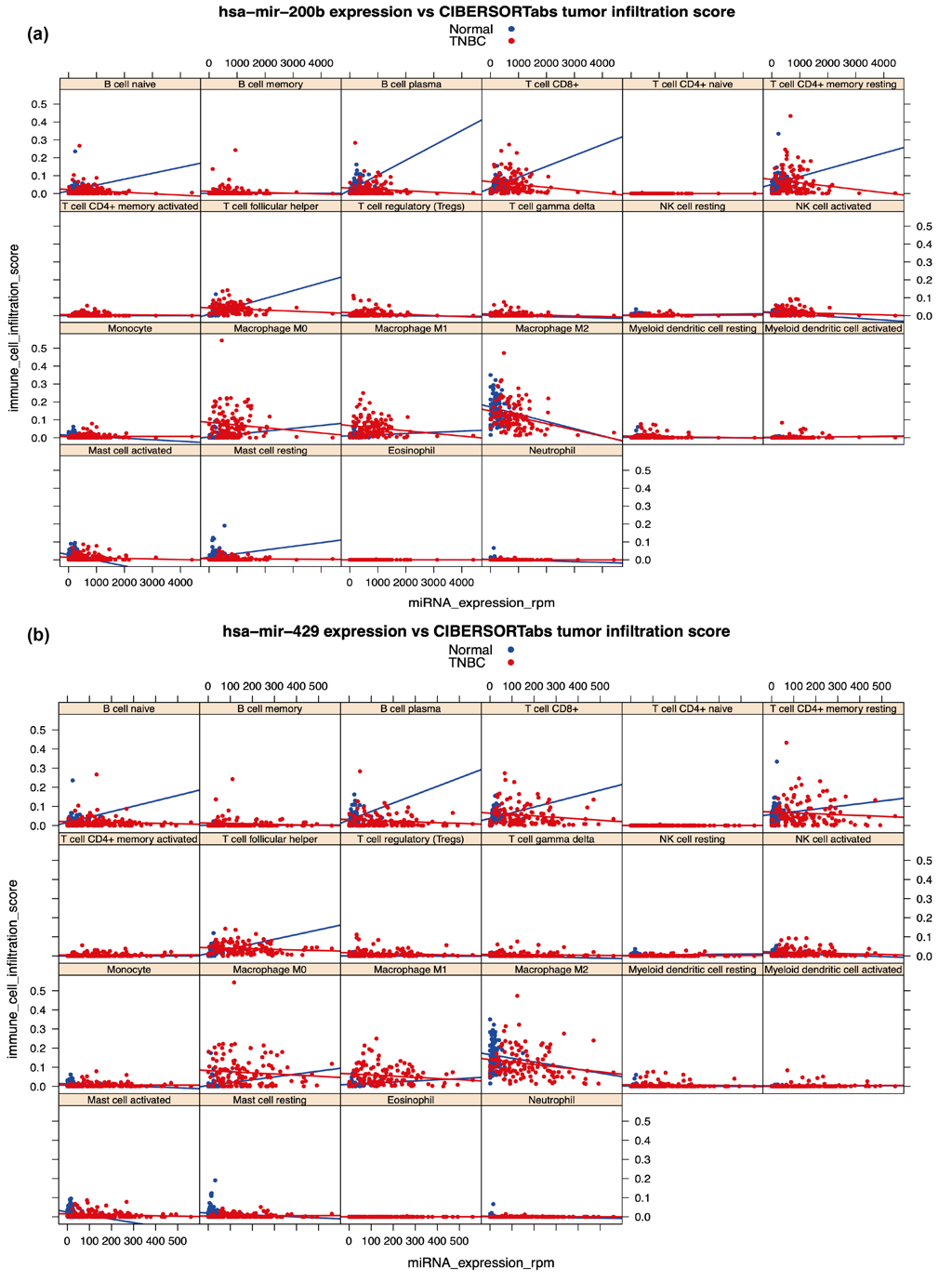


**Figure S8:** **Tumour infiltration analysis of signature miRNAs:** (a) Tumour infiltration score analysis using CIBERSORT-abs score for miR-200b between normal and TNBC samples. (b) Tumour infiltration score analysis using CIBERSORT-abs score for miR-429 between normal TNBC samples.

(a)

(b)

(c)

(d)

**Figure S9:** **Tumour infiltration analysis of signature miRNAs:** Tumour infiltration score analysis using CIBERSORT-abs score for (a) miR-17, (b) miR-20a, (c) miR-93, and (d) miR-106b between normal and TNBC samples.

Table S1: *In-silico* predicted list of microRNAs targeting HR pathway genes

| Sl. No. | HR pathway genes | Description | Targeting microRNAs (In-silico) |
| --- | --- | --- | --- |
| 1 | *AP5S1* | Essential for efficient homologous recombination | hsa-miR-452-3p |
| 2 | *BABAM1* | BRISC and BRCA1 A complex member 1 | hsa-miR-502-5p |
| 3 | *BABAM2* | BRISC and BRCA1 A complex member 2 | hsa-miR-6875-5p |
| 4 | *BLM* | BLM RecQ like helicase | hsa-miR-628-5p |
| 5 | *BRCA1* | Plays central role in the HR by forming complex with BRCA2 and RAD51 proteins | hsa-miR-125a-3p, hsa-miR-183-5p, hsa-miR-212-3p, hsa-miR-132-3p |
| 6 | *BRIP1* | Unwinds secondary DNA structures | hsa-miR-616-5p |
| 7 | *EME1* | Essential meiotic structure-specific endonuclease 1 | hsa-miR-876-5p |
| 8 | *EXD2* | Binds to MRN complex and enhances DSB end resection | hsa-miR-218-5p, hsa-miR-6783-3p |
| 9 | *GEN1* | Resolves Holiday Junctions | hsa-miR-6728-3p |
| 10 | *LRATD2* | Homologous strand exchange | hsa-let-7b-5p, hsa-miR-17-5p, hsa-miR-27a-3p, hsa-miR-197-3p |
| 11 | *MORF4L1* | Required for association of BRCA2, PALB2 and RAD51 with DNA | hsa-miR-20a-5p, hsa-miR-106a-5p, hsa-miR-1271-5p, hsa-miR-182-5p, hsa-miR-29b-3p, hsa-miR-29a-3p, hsa-miR-29c-3p, hsa-miR-33b-5p, hsa-miR-330-3p, hsa-miR-676-5p |
| 12 | *MRE11* | MRE11 homolog, double strand break repair nuclease | hsa-miR-6807-3p |
| 13 | *PPP4C* | Protein phosphatase 4 catalytic subunit | hsa-miR-4640-5p, hsa-miR-4726-5p |
| 14 | *PPP4R1* | Dephosphorylates RPA2 | hsa-miR-7-5p |
| 15 | *PPP4R2* | Dephosphorylates RPA2 | hsa-miR-200c-3p, hsa-miR-429, hsa-miR-200b-3p |
| 16 | *PPP4R3A* | Mediates dephosphorylation of RPA2 required for recruitment of RAD51 | hsa-miR-125a-5p, hsa-miR-125b-5p, hsa-miR-96-5p, hsa-miR-1271-5p |
| 17 | *PPP4R4* | Protein phosphatase 4 regulatory subunit 4 | hsa-miR-23a-3p |
| 18 | *RAD50* | RAD50 double strand break repair protein | hsa-miR-539-5p, hsa-miR-195-5p |
| 19 | *RAD51* | Recruited at DSB sites to facilitate HR along with BRCA1/2 and other RAD51 family proteins | hsa-miR-20a-3p |
| 20 | *RAD52* | Binds to DNA nicks at DSB sites to recruit BRCA1/2 and RAD51 complex proteins | hsa-miR-758-3p |
| 21 | *RAD54B* | RAD54 homolog B | hsa-miR-942-5p, miR-139 |
| 22 | *RAD54L2* | Facilitates homologous pairing | hsa-miR-616-5p, hsa-miR-132-3p, hsa-miR-7-5p |
| 23 | *RBBP8* | DSB end resection | hsa-miR-130b-3p, hsa-miR-19a-3p, hsa-miR-19b-3p, hsa-miR-3129-3p, hsa-miR-301b-3p, hsa-miR-301a-3p, hsa-miR-130a-3p, hsa-miR-454-3p |
| 24 | *RMI1* | Dissolution of recombination intermediates | hsa-miR-495-3p, hsa-miR-362-5p |
| 25 | *RPA1* | Stabilizes ssDNA intermediates | hsa-miR-452-5p, hsa-miR-345-5p, hsa-miR-5001-3p |
| 26 | *RPA2* | Stabilizes ssDNA intermediates | hsa-miR-130a-3p, hsa-miR-454-3p, hsa-miR-301a-3p, hsa-miR-301b-3p |
| 27 | *RPA3* | Stabilizes ssDNA intermediates | hsa-miR-7705 |
| 28 | *RPA4* | Interacts with RAD51 for strand exchange | hsa-miR-330-3p |
| 29 | *SAMHD1* | DNA end resection | hsa-miR-181c-5p, hsa-miR-181b-5p, hsa-miR-181a-5p, hsa-miR-181d-5p, hsa-miR-30c-5p, hsa-miR-30b-5p |
| 30 | *SEM1* | Stabilizes BRCA2 | hsa-miR-6728-3p, hsa-miR-18a-3p |
| 31 | *SMC5* | Recruits cohesin to sites of DSB | hsa-miR-495-3p, hsa-miR-3613-3p |
| 32 | *SMC6* | Recruits cohesin to sites of DSB | hsa-miR-153-5p, hsa-miR-579-3p |
| 33 | *TOPBP1* | Interacts with 53BP1 and BRCA1 | hsa-miR-182-5p, hsa-miR-33a-3p |
| 34 | *UIMC1* | Specifically binds to Lys-63 linked ubiquitinated histones H2A and H2AX at DSB | hsa-miR-138-5p |
| 35 | *WRN* | Interacts with MRN complex; Resolution of recombination intermediates | hsa-miR-877-3p, hsa-miR-1276 |
| 36 | *XRCC2* | Binds to double strand breaks and facilitates HR | hsa-miR-10a-5p |
| 37 | *XRCC3* | Binds to double strand breaks and facilitates HR | hsa-miR-6764-5p, hsa-miR-1915-3p |
| 38 | *ZFYVE26* | Essential for efficient homologous recombination | hsa-miR-106b-5p, hsa-miR-20a-5p, hsa-miR-20b-5p, hsa-miR-93-5p, hsa-miR-17-5p, hsa-let-7a-5p, hsa-miR-218-5p, hsa-miR-106a-5p, hsa-miR-148a-3p, hsa-miR-152-3p, hsa-miR-148b-3p |

Table S2: *In-silico* predicted list of microRNAs upon Pearson’s correlation analysis

| Sl. No. | microRNAs | Target Genes in HR pathway | Pearson correlation coefficient | P-value |
| --- | --- | --- | --- | --- |
| 1 | hsa-miR-106b | *ZFYVE26* | -0.2852 | 0.002 |
| 2 | hsa-miR-93 | *ZFYVE26* | -0.2815 | 0.002 |
| 3 | hsa-miR-182 | *MORF4L1* | -0.2806 | 0.002 |
| 4 | hsa-miR-181c | *SAMHD1* | -0.2744 | 0.003 |
| 5 | hsa-miR-181b | *SAMHD1* | -0.2754 | 0.003 |
| 6 | hsa-miR-181d | *SAMHD1* | -0.2762 | 0.003 |
| 7 | hsa-miR-139 | *RAD54B* | -0.2676 | 0.003 |
| 8 | hsa-miR-616 | *RAD54L2* | 0.2626 | 0.004 |
| 9 | hsa-miR-20a | *ZFYVE26* | -0.2649 | 0.004 |
| 10 | hsa-miR-17 | *ZFYVE26* | -0.2492 | 0.006 |
| 11 | hsa-miR-181a | *SAMHD1* | -0.2349 | 0.011 |
| 12 | hsa-miR-19b | *RBBP8* | 0.2288 | 0.013 |
| 13 | hsa-miR-200b | *PPP4R2* | -0.226 | 0.014 |
| 14 | hsa-miR-429 | *PPP4R2* | -0.2128 | 0.021 |
| 15 | hsa-miR-130b | *RBBP8* | 0.1848 | 0.045 |
| 16 | hsa-miR-495 | *RMI1* | -0.1598 | 0.088 |
| 17 | hsa-miR-330 | *MORF4L1* | 0.1553 | 0.093 |
| 18 | hsa-miR-6875 | *BABAM2* | -0.1545 | 0.095 |
| 19 | hsa-miR-148a | *ZFYVE26* | 0.1499 | 0.105 |
| 20 | hsa-miR-10a | *XRCC2* | -0.1453 | 0.117 |
| 21 | hsa-miR-19a | *RBBP8* | 0.1399 | 0.131 |
| 22 | hsa-miR-200c | *PPP4R2* | -0.1374 | 0.138 |
| 23 | hsa-miR-138 | *UIMC1* | 0.1331 | 0.151 |
| 24 | hsa-miR-20a | *MORF4L1* | -0.126 | 0.174 |
| 25 | hsa-miR-30c | *SAMHD1* | -0.1235 | 0.183 |
| 26 | hsa-miR-301a | *RBBP8* | 0.1067 | 0.252 |
| 27 | hsa-miR-29b | *MORF4L1* | -0.1052 | 0.257 |
| 28 | hsa-miR-33b | *MORF4L1* | -0.0878 | 0.344 |
| 29 | hsa-miR-3613 | *SMC5* | -0.07174 | 0.44 |
| 30 | hsa-miR-106a | *ZFYVE26* | -0.07148 | 0.444 |
| 31 | hsa-miR-301b | *RBBP8* | -0.09725 | 0.2927 |
| 32 | hsa-miR-301b | *RPA2* | 0.06747 | 0.468 |
| 33 | hsa-miR-20b | *ZFYVE26* | -0.05556 | 0.555 |
| 34 | hsa-miR-96 | *PPP4R3A* | 0.05082 | 0.585 |
| 35 | hsa-miR-454 | *RPA2* | -0.04817 | 0.604 |
| 36 | hsa-miR-153 | *SMC6* | 0.0435 | 0.64 |
| 37 | hsa-miR-5001 | *RPA1* | 0.04002 | 0.667 |
| 38 | hsa-miR-877 | *WRN* | 0.03732 | 0.688 |
| 39 | hsa-miR-7 | *RAD54L2* | -0.02538 | 0.785 |
| 40 | hsa-miR-454 | *RBBP8* | 0.01884 | 0.786 |
| 41 | hsa-miR-1276 | *WRN* | -0.02233 | 0.81 |
| 42 | hsa-miR-345 | *RPA1* | -0.02137 | 0.818 |
| 43 | hsa-miR-6807 | *MRE11* | -0.01306 | 0.889 |
| 44 | hsa-miR-6783 | *EXD2* | -0.008499 | 0.927 |
| 45 | hsa-miR-197 | *LRATD2* | -0.00844 | 0.928 |
| 46 | hsa-miR-106a | *MORF4L1* | 0.006496 | 0.945 |
| 47 | hsa-miR-301a | *RPA2* | 0.006202 | 0.947 |
| 48 | hsa-miR-27a | *LRATD2* | -0.003217 | 0.972 |
| 49 | hsa-miR-17 | *LRATD2* | 0.002251 | 0.981 |
| 50 | hsa-miR-148b | *ZFYVE26* | -0.002055 | 0.982 |

Table S3: Validated list of microRNAs upon Pearson’s correlation analysis

| Sl. No. | microRNAs | HR-Target Genes | Pearson correlation coefficient | P-value |
| --- | --- | --- | --- | --- |
| 1 | hsa-miR-155 | *SAMHD1* | 0.2764 | 0.002 |
| 2 | hsa-miR-181a | *SAMHD1* | -0.2349 | 0.011 |
| 3 | hsa-miR-127 | *XRCC3* | -0.1806 | 0.05 |
| 4 | hsa-miR-34a | *RAD51* | -0.1386 | 0.134 |
| 5 | hsa-miR-210 | *RAD52* | -0.1225 | 0.186 |
| 6 | hsa-miR-125a | *BRCA1* | -0.04511 | 0.628 |
| 7 | hsa-miR-193b | *RAD51* | 0.04492 | 0.629 |

Table S4: Final list of miRNA/target genes selected for biomarker validation

| Sl. No. | microRNAs | HR-Target Genes | Pearson correlation coefficient | P-value |
| --- | --- | --- | --- | --- |
| 1 | hsa-miR-106b | *ZFYVE26* | -0.2852 | 0.002 |
| 2 | hsa-miR-93 | *ZFYVE26* | -0.2815 | 0.002 |
| 3 | hsa-miR-182 | *MORF4L1* | -0.2806 | 0.002 |
| 4 | hsa-miR-181c | *SAMHD1* | -0.2744 | 0.003 |
| 5 | hsa-miR-181b | *SAMHD1* | -0.2754 | 0.003 |
| 6 | hsa-miR-181d | *SAMHD1* | -0.2762 | 0.003 |
| 7 | hsa-miR-139 | *RAD54B* | -0.2676 | 0.003 |
| 8 | hsa-miR-20a | *ZFYVE26* | -0.2649 | 0.004 |
| 9 | hsa-miR-17 | *ZFYVE26* | -0.2492 | 0.006 |
| 10 | hsa-miR-181a | *SAMHD1* | -0.2349 | 0.011 |
| 11 | hsa-miR-200b | *PPP4R2* | -0.226 | 0.014 |
| 12 | hsa-miR-429 | *PPP4R2* | -0.2128 | 0.021 |
| 13 | hsa-miR-127 | *XRCC3* | -0.1806 | 0.05 |
